# Supplementary material for: Hybrid Models and Biological Model Reduction with PyDSTool
Source: PLoS Comput Biol. 2012 Aug 9;8(8):e1002628. doi: 10.1371/journal.pcbi.1002628 (PMC3415397; doi:10.1371/journal.pcbi.1002628)
Supplement: Text S4 — Complete source code for the PyDSTool package (version 0.88.120504). Includes API documentation and help files linking to web pages. This file is identical to the current public release on Sourceforge.net. (ZIP) [file pcbi.1002628.s004.zip › PyDSTool/html/PyDSTool.Toolbox.optimizers.helpers.levenberg_marquardt-pysrc.html]

xml version="1.0" encoding="ascii"?


PyDSTool.Toolbox.optimizers.helpers.levenberg\_marquardt


| Home | Trees | Indices | Help | PyDSTool |
| --- | --- | --- | --- | --- |

Package PyDSTool ::
Package Toolbox ::
Package optimizers ::
Package helpers ::
Module levenberg\_marquardt

[hide private]

[frames] | no frames]

# Source Code for Module PyDSTool.Toolbox.optimizers.helpers.levenberg\_marquardt

```
 1   
 2  # Matthieu Brucher 
 3  # Last Change : 2007-08-23 10:12 
 4   
 5  import quadratic 
 6  import numpy 
 7   


8 -class LMQuadratic(quadratic.Quadratic):


9    """ 
10    Defines a cost function with a quadratic cost but the Levenberg-Marquardt approximation of the hessian 
11    """ 


12 -  def hessian(self, params):


13      """ 
14      Compute sthe hessian of the fit function 
15      """ 
16      inter = 2 * self.f.gradient(self.x, params)[..., numpy.newaxis] * self.f.gradient(self.x, params)[..., numpy.newaxis, :] 
17      shape = inter.shape[-2], inter.shape[-1] 
18      inter.shape = (-1, inter.shape[-2] * inter.shape[-1]) 
19      temp = numpy.sum(inter, axis = 0) 
20      temp.shape = shape 
21      return temp

22
```

  


| Home | Trees | Indices | Help | PyDSTool |
| --- | --- | --- | --- | --- |

|  |  |
| --- | --- |
| Generated by Epydoc 3.0.1 on Fri May 4 15:24:12 2012 | http://epydoc.sourceforge.net |
